# Supplementary material for: Carbogen inhalation during non-convulsive status epilepticus: A quantitative exploratory analysis of EEG recordings
Source: PLoS One. 2021 Feb 3;16(2):e0240507. doi: 10.1371/journal.pone.0240507 (PMC7857554; doi:10.1371/journal.pone.0240507)
Supplement: S4 Fig — (DOCX) [file pone.0240507.s004.docx]

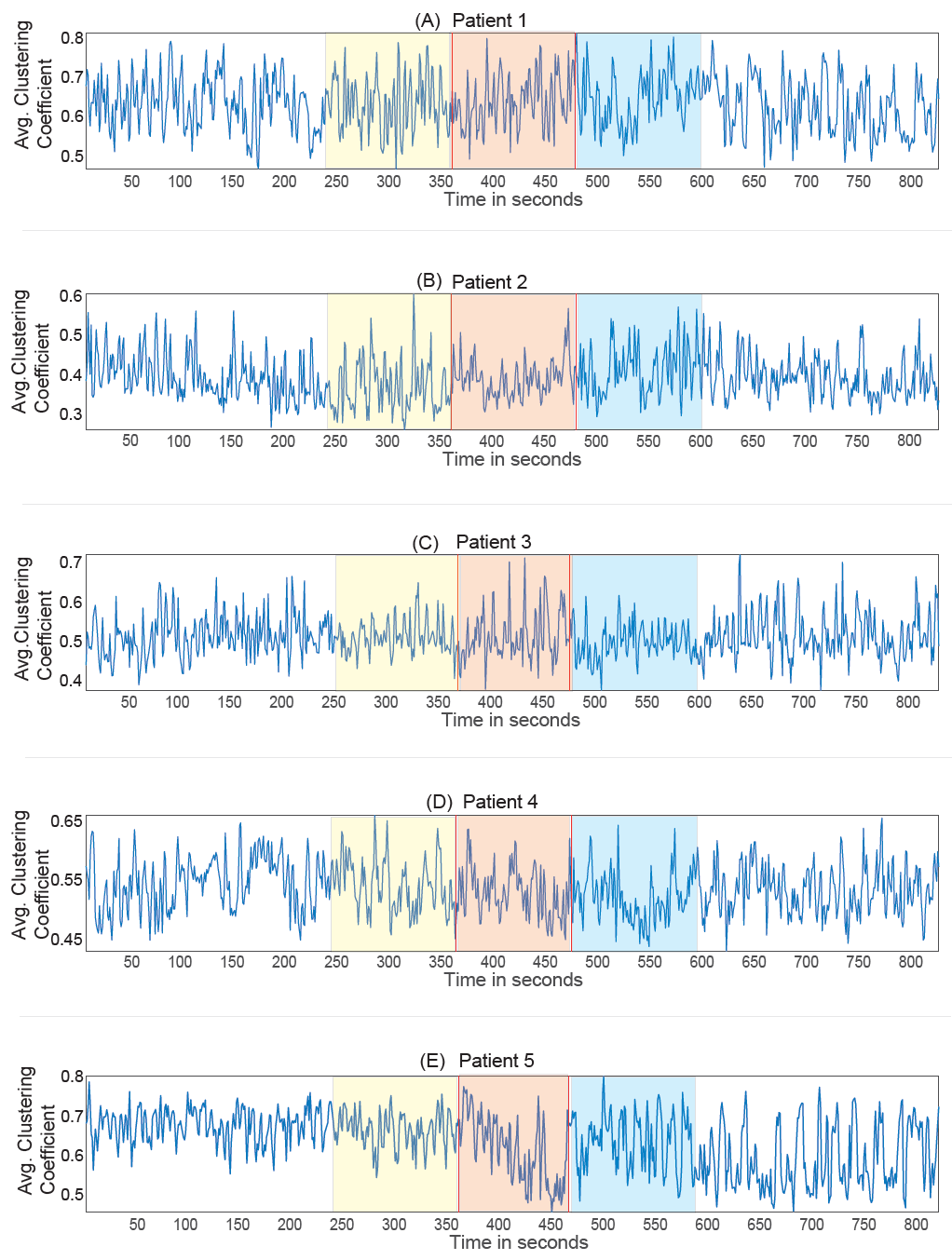


**S4 Fig. Non-smoothed clustering coefficient time series in broadband across three different states for five patients.**
