# Supplementary material for: Carbogen inhalation during non-convulsive status epilepticus: A quantitative exploratory analysis of EEG recordings
Source: PLoS One. 2021 Feb 3;16(2):e0240507. doi: 10.1371/journal.pone.0240507 (PMC7857554; doi:10.1371/journal.pone.0240507)
Supplement: S5 Fig — The normalised percentage change in band power across different frequencies between A) “Before-During” state and B) “Before-After” state. (DOCX) [file pone.0240507.s005.docx]

**A B**


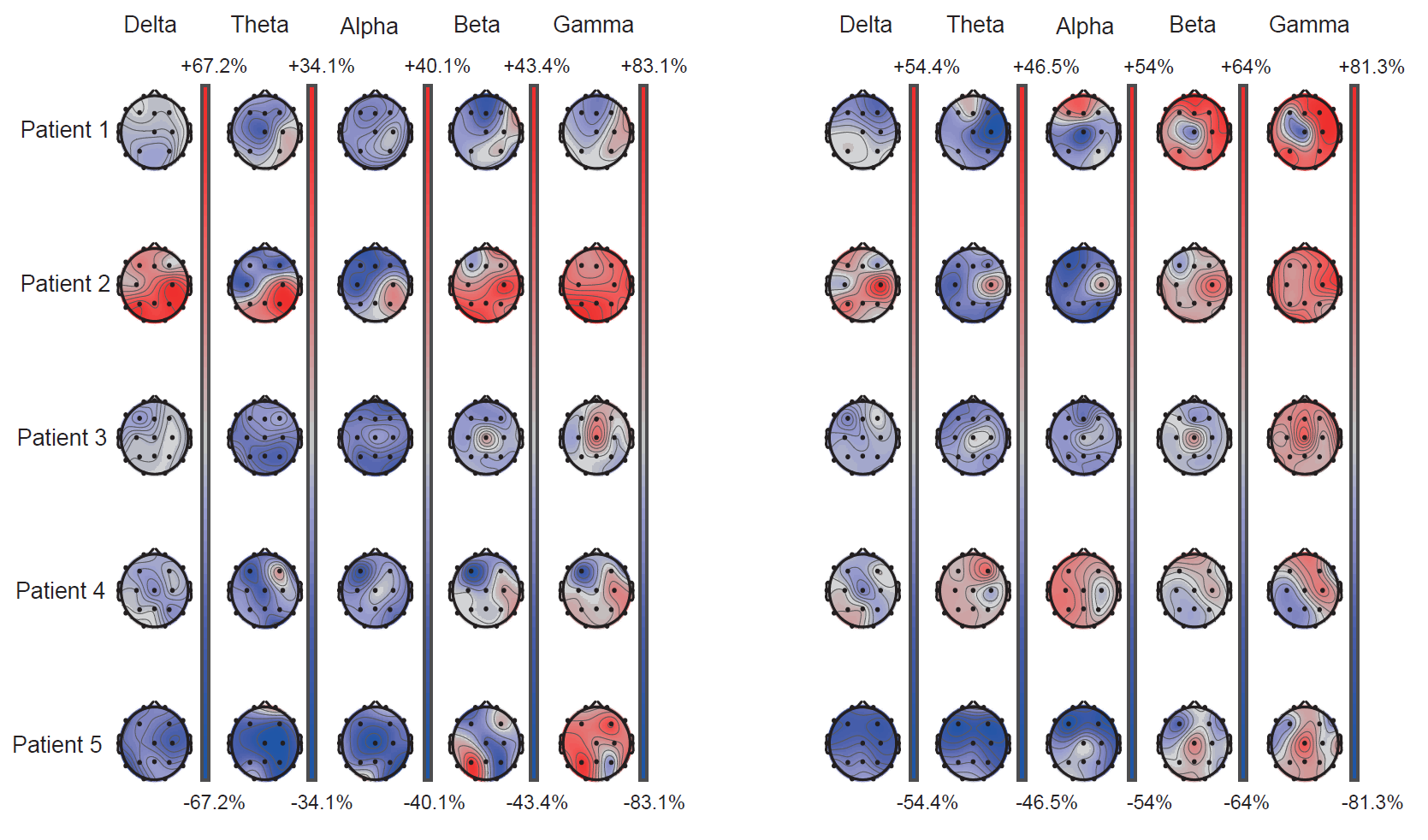


**S5 Fig. Percentage change in band power.** The normalised percentage change in band power across different frequencies between A) “Before-During” state and B) “Before-After” state.
