# Supplementary material for: Carbogen inhalation during non-convulsive status epilepticus: A quantitative exploratory analysis of EEG recordings
Source: PLoS One. 2021 Feb 3;16(2):e0240507. doi: 10.1371/journal.pone.0240507 (PMC7857554; doi:10.1371/journal.pone.0240507)
Supplement: S7 Fig — (A)-(E) Sub-band normalised correlation coefficient time series for Patient 1-Patient 5. (F)-(H) Broandband average time series for Patient 1, Patient 2 and Patient 4. (DOCX) [file pone.0240507.s007.docx]

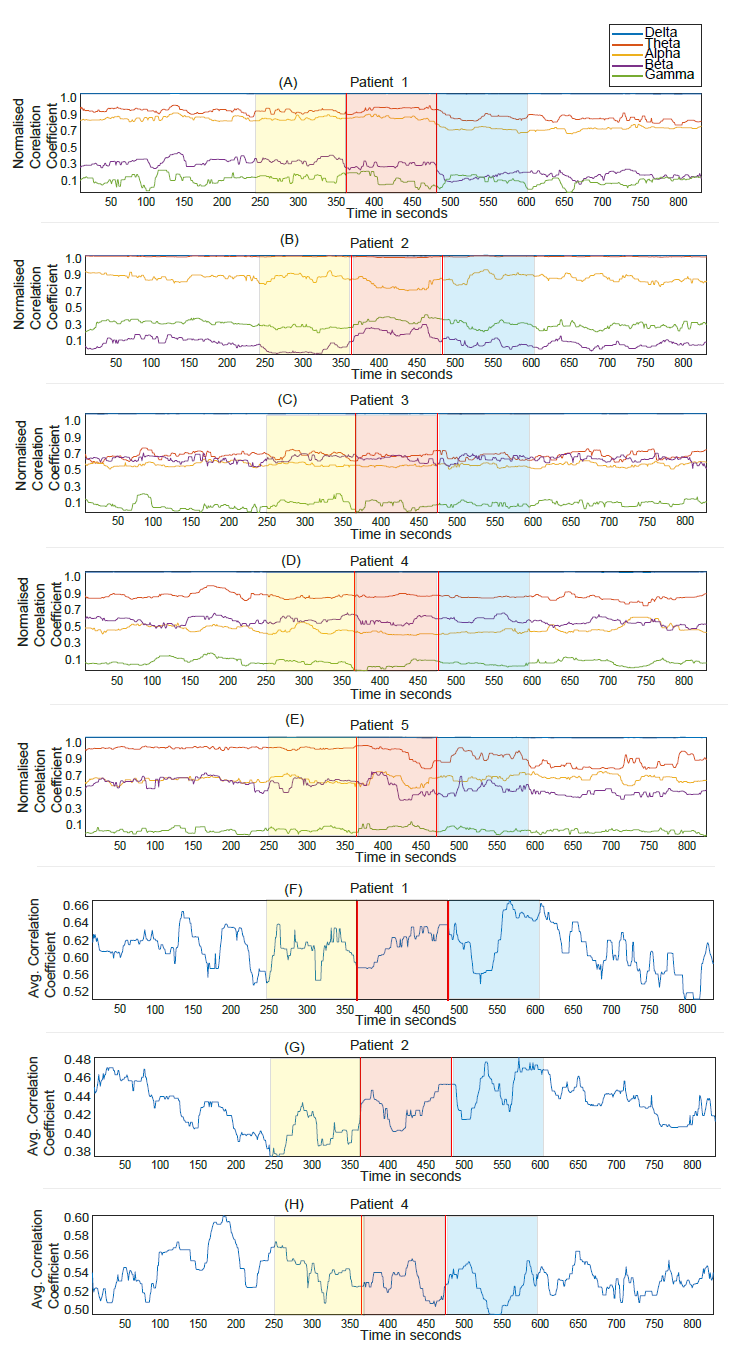


**S7 Fig. Correlation coefficient time series.** (A)-(E) Sub-band normalised correlation coefficient time series for Patient 1-Patient 5. (F)-(H) Broandband average time series for Patient 1, Patient 2 and Patient 4.
