# Supplementary material for: Carbogen inhalation during non-convulsive status epilepticus: A quantitative exploratory analysis of EEG recordings
Source: PLoS One. 2021 Feb 3;16(2):e0240507. doi: 10.1371/journal.pone.0240507 (PMC7857554; doi:10.1371/journal.pone.0240507)
Supplement: S1 Table — (DOCX) [file pone.0240507.s010.docx]

| Channel | Before-During | | | | | Before-After | | | | |
| --- | --- | --- | --- | --- | --- | --- | --- | --- | --- | --- |
|  | **Delta** | **Theta** | **Alpha** | **Beta** | **Gamma** | **Delta** | **Theta** | **Alpha** | **Beta** | **Gamma** |
| 'C4' | 0.19 | -0.05 | 0.08 | 0.00 | -0.20 | 0.40 | 1.09 | 0.28 | -0.93 | -0.50 |
| 'CZ' | 0.25 | 0.57 | 0.29 | 0.54 | 0.68 | 0.18 | 0.81 | 1.14 | 0.86 | 1.16 |
| 'F3' | 0.13 | 0.31 | 0.36 | 0.28 | 0.37 | 0.59 | 0.19 | -0.43 | -0.30 | 0.26 |
| 'F4' | 0.15 | 0.33 | 0.27 | 0.32 | 0.24 | 0.84 | 0.79 | 0.11 | -0.47 | -0.29 |
| 'F7' | 0.15 | 0.11 | 0.24 | 0.18 | 0.13 | 0.78 | 0.45 | -0.26 | -0.71 | -0.42 |
| 'F8' | 0.04 | 0.11 | 0.19 | -0.13 | -0.14 | 0.74 | 0.88 | 0.05 | -0.99 | -0.55 |
| 'FZ' | 0.20 | 0.33 | 0.37 | 0.29 | 0.28 | 0.83 | -0.06 | -0.55 | -0.58 | -0.43 |
| 'FP1' | 0.15 | 0.12 | 0.27 | 0.25 | 0.26 | 0.79 | -0.14 | -0.80 | -0.90 | -0.51 |
| 'FP2' | 0.15 | 0.21 | 0.25 | 0.25 | 0.24 | 1.09 | 0.29 | -0.43 | -0.75 | -0.50 |
| 'O1' | 0.43 | 0.05 | 0.41 | 0.09 | 0.01 | 0.13 | 0.24 | 0.34 | -0.46 | -0.48 |
| 'O2' | 0.34 | -0.08 | 0.40 | 0.15 | 0.07 | 0.11 | 0.50 | 0.11 | -0.54 | -0.51 |
| 'P3' | 0.36 | 0.17 | 0.27 | 0.13 | 0.10 | -0.06 | 0.25 | 0.49 | -0.61 | -0.52 |
| 'P4' | 0.35 | -0.04 | 0.13 | -0.07 | -0.11 | 0.13 | 0.54 | 0.03 | -0.61 | -0.53 |
| 'T3' | 0.28 | 0.15 | 0.25 | 0.14 | 0.02 | 0.12 | 0.33 | 0.50 | -0.36 | -0.51 |
| 'T4' | 0.05 | -0.02 | 0.35 | -0.05 | -0.13 | 0.32 | 0.80 | 0.35 | -0.88 | -0.61 |
| 'T5' | 0.27 | 0.00 | 0.44 | 0.15 | -0.04 | 0.06 | 0.19 | 0.49 | -0.34 | -0.47 |
| 'T6' | 0.02 | -0.10 | 0.42 | 0.20 | 0.01 | -0.01 | 0.59 | 0.24 | -0.42 | -0.46 |

**S1 Table.** Patient 1 Effect size (Effect size *d-*values) for all the channels across all the frequency bands for before-during and before-after state.
