# Supplementary material for: Carbogen inhalation during non-convulsive status epilepticus: A quantitative exploratory analysis of EEG recordings
Source: PLoS One. 2021 Feb 3;16(2):e0240507. doi: 10.1371/journal.pone.0240507 (PMC7857554; doi:10.1371/journal.pone.0240507)
Supplement: S2 Table — (DOCX) [file pone.0240507.s011.docx]

| Channel | Before-During | | | | | Before-After | | | | |
| --- | --- | --- | --- | --- | --- | --- | --- | --- | --- | --- |
|  | **Delta** | **Theta** | **Alpha** | **Beta** | **Gamma** | **Delta** | **Theta** | **Alpha** | **Beta** | **Gamma** |
| 'C4' | 0.274 | 0.803 | 0.685 | 1.000 | 0.180 | 0.004 | 0.000 | 0.039 | 0.000 | 0.000 |
| 'CZ' | 0.126 | 0.002 | 0.080 | 0.002 | 0.002 | 0.188 | 0.000 | 0.000 | 0.000 | 0.000 |
| 'F3' | 0.484 | 0.062 | 0.030 | 0.043 | 0.002 | 0.000 | 0.159 | 0.002 | 0.016 | 0.050 |
| 'F4' | 0.403 | 0.047 | 0.096 | 0.044 | 0.111 | 0.000 | 0.000 | 0.451 | 0.000 | 0.000 |
| 'F7' | 0.403 | 0.564 | 0.140 | 0.370 | 0.496 | 0.000 | 0.000 | 0.056 | 0.000 | 0.000 |
| 'F8' | 0.835 | 0.541 | 0.275 | 0.550 | 0.476 | 0.000 | 0.000 | 0.737 | 0.000 | 0.000 |
| 'FZ' | 0.235 | 0.047 | 0.030 | 0.024 | 0.026 | 0.000 | 0.678 | 0.000 | 0.000 | 0.000 |
| 'FP1' | 0.427 | 0.496 | 0.096 | 0.078 | 0.049 | 0.000 | 0.338 | 0.000 | 0.000 | 0.000 |
| 'FP2' | 0.403 | 0.220 | 0.111 | 0.075 | 0.111 | 0.000 | 0.029 | 0.000 | 0.000 | 0.000 |
| 'O1' | 0.013 | 0.798 | 0.016 | 0.679 | 0.965 | 0.351 | 0.080 | 0.013 | 0.001 | 0.000 |
| 'O2' | 0.038 | 0.685 | 0.014 | 0.437 | 0.745 | 0.452 | 0.000 | 0.417 | 0.000 | 0.000 |
| 'P3' | 0.032 | 0.373 | 0.096 | 0.488 | 0.583 | 0.656 | 0.059 | 0.000 | 0.000 | 0.000 |
| 'P4' | 0.038 | 0.863 | 0.488 | 0.713 | 0.550 | 0.361 | 0.000 | 0.854 | 0.000 | 0.000 |
| 'T3' | 0.096 | 0.403 | 0.118 | 0.488 | 0.935 | 0.396 | 0.016 | 0.000 | 0.005 | 0.000 |
| 'T4' | 0.803 | 0.935 | 0.030 | 0.874 | 0.635 | 0.017 | 0.000 | 0.008 | 0.000 | 0.000 |
| 'T5' | 0.096 | 0.987 | 0.010 | 0.485 | 0.935 | 0.651 | 0.160 | 0.000 | 0.007 | 0.000 |
| 'T6' | 0.945 | 0.588 | 0.010 | 0.182 | 0.965 | 0.946 | 0.000 | 0.075 | 0.000 | 0.000 |

**S2 Table.** Patient 1 Permutation test p-values (FDR corrected) for all the channels across all frequency sub-bands in before-during and before-after state.
