# Supplementary material for: Carbogen inhalation during non-convulsive status epilepticus: A quantitative exploratory analysis of EEG recordings
Source: PLoS One. 2021 Feb 3;16(2):e0240507. doi: 10.1371/journal.pone.0240507 (PMC7857554; doi:10.1371/journal.pone.0240507)
Supplement: S3 Table — (DOCX) [file pone.0240507.s012.docx]

| Channel | Before-During | | | | | Before-After | | | | |
| --- | --- | --- | --- | --- | --- | --- | --- | --- | --- | --- |
|  | **Delta** | **Theta** | **Alpha** | **Beta** | **Gamma** | **Delta** | **Theta** | **Alpha** | **Beta** | **Gamma** |
| 'Fp1' | -0.59 | 0.57 | 0.77 | 0.19 | -0.44 | -0.50 | 0.38 | 1.06 | 0.14 | -0.57 |
| 'Fp2' | -0.57 | 0.13 | 0.37 | -0.92 | -0.82 | -0.21 | 0.06 | 0.66 | -0.67 | -0.94 |
| 'F3' | -0.73 | 0.16 | 0.61 | 0.31 | -0.41 | -0.36 | 0.51 | 1.04 | 0.50 | -0.44 |
| 'F4' | -0.03 | 0.47 | 0.55 | -0.15 | -0.41 | 0.26 | 0.46 | 0.55 | -0.17 | -0.58 |
| 'C3' | -0.16 | 0.56 | 0.86 | -0.30 | -0.30 | 0.22 | 0.70 | 0.86 | -0.33 | -0.48 |
| 'C4' | -1.42 | -0.62 | -0.28 | -1.50 | -0.86 | -0.98 | -0.42 | -0.24 | -1.72 | -1.25 |
| 'P3' | -1.30 | -0.21 | 0.31 | -0.74 | -0.91 | -0.76 | 0.52 | 0.65 | -0.29 | -0.39 |
| 'P4' | -1.33 | -0.48 | -0.21 | -0.57 | -0.78 | -0.23 | 0.40 | 0.73 | -0.43 | -0.37 |
| 'O1' | -1.29 | -0.29 | 0.08 | -1.02 | -1.26 | -0.21 | 0.65 | 0.90 | -0.81 | -1.29 |
| 'O2' | -1.31 | -0.29 | 0.06 | -0.83 | -1.12 | -0.01 | 0.54 | 0.96 | -0.51 | -0.60 |
| 'F7' | -0.43 | 0.52 | 0.63 | -0.34 | -0.70 | -0.36 | 0.49 | 0.84 | -0.16 | -0.65 |
| 'F8' | -0.10 | 0.40 | 0.63 | -0.59 | -0.94 | 0.05 | 0.28 | 0.75 | -0.32 | -0.50 |
| 'T3' | -0.23 | 0.40 | 0.49 | -0.70 | -0.70 | 0.16 | 0.56 | 0.62 | -0.68 | -0.83 |
| 'T4' | -0.91 | -0.18 | 0.24 | -0.94 | -1.10 | -0.12 | 0.10 | 0.36 | -0.81 | -1.26 |
| 'T5' | -1.23 | -0.18 | 0.25 | -0.96 | -1.24 | -0.48 | 0.67 | 0.85 | -0.85 | -1.13 |
| 'T6' | -1.44 | -0.47 | -0.17 | -0.47 | -0.49 | -0.17 | 0.61 | 0.98 | -0.17 | -0.17 |
| 'Fz' | -0.57 | 0.33 | 0.65 | -0.09 | -0.42 | -0.05 | 0.33 | 0.82 | -0.09 | -0.69 |
| 'Pz' | -1.04 | -0.30 | 0.05 | -0.71 | -0.88 | -0.34 | 0.45 | 0.57 | -0.29 | -0.68 |

**S3 Table.** Patient 2 Effect size (Effect size *d-*values) for all the channels across all the frequency bands for before-during and before-after state.
