# Supplementary material for: Carbogen inhalation during non-convulsive status epilepticus: A quantitative exploratory analysis of EEG recordings
Source: PLoS One. 2021 Feb 3;16(2):e0240507. doi: 10.1371/journal.pone.0240507 (PMC7857554; doi:10.1371/journal.pone.0240507)
Supplement: S4 Table — (DOCX) [file pone.0240507.s013.docx]

| Channel | Before-During | | | | | Before-After | | | | |
| --- | --- | --- | --- | --- | --- | --- | --- | --- | --- | --- |
|  | **Delta** | **Theta** | **Alpha** | **Beta** | **Gamma** | **Delta** | **Theta** | **Alpha** | **Beta** | **Gamma** |
| 'Fp1' | 0.000 | 0.000 | 0.000 | 0.161 | 0.000 | 0.000 | 0.006 | 0.000 | 0.327 | 0.000 |
| 'Fp2' | 0.000 | 0.335 | 0.006 | 0.000 | 0.000 | 0.122 | 0.674 | 0.000 | 0.000 | 0.000 |
| 'F3' | 0.000 | 0.237 | 0.000 | 0.020 | 0.001 | 0.008 | 0.001 | 0.000 | 0.000 | 0.001 |
| 'F4' | 0.831 | 0.000 | 0.000 | 0.261 | 0.000 | 0.057 | 0.001 | 0.000 | 0.224 | 0.000 |
| 'C3' | 0.259 | 0.000 | 0.000 | 0.005 | 0.013 | 0.104 | 0.000 | 0.000 | 0.014 | 0.000 |
| 'C4' | 0.000 | 0.000 | 0.036 | 0.000 | 0.000 | 0.000 | 0.002 | 0.078 | 0.000 | 0.000 |
| 'P3' | 0.000 | 0.114 | 0.021 | 0.000 | 0.000 | 0.000 | 0.000 | 0.000 | 0.032 | 0.002 |
| 'P4' | 0.000 | 0.000 | 0.125 | 0.000 | 0.000 | 0.093 | 0.002 | 0.000 | 0.002 | 0.004 |
| 'O1' | 0.000 | 0.028 | 0.569 | 0.000 | 0.000 | 0.114 | 0.000 | 0.000 | 0.000 | 0.000 |
| 'O2' | 0.000 | 0.028 | 0.678 | 0.000 | 0.000 | 0.925 | 0.000 | 0.000 | 0.000 | 0.000 |
| 'F7' | 0.002 | 0.000 | 0.000 | 0.009 | 0.000 | 0.007 | 0.000 | 0.000 | 0.242 | 0.000 |
| 'F8' | 0.450 | 0.004 | 0.000 | 0.000 | 0.000 | 0.699 | 0.035 | 0.000 | 0.018 | 0.000 |
| 'T3' | 0.081 | 0.004 | 0.000 | 0.000 | 0.000 | 0.226 | 0.000 | 0.000 | 0.000 | 0.000 |
| 'T4' | 0.000 | 0.206 | 0.072 | 0.000 | 0.000 | 0.379 | 0.482 | 0.008 | 0.000 | 0.000 |
| 'T5' | 0.000 | 0.179 | 0.068 | 0.000 | 0.000 | 0.001 | 0.000 | 0.000 | 0.000 | 0.000 |
| 'T6' | 0.000 | 0.000 | 0.206 | 0.000 | 0.000 | 0.219 | 0.000 | 0.000 | 0.215 | 0.231 |
| 'Fz' | 0.000 | 0.013 | 0.000 | 0.485 | 0.000 | 0.699 | 0.011 | 0.000 | 0.493 | 0.000 |
| 'Pz' | 0.000 | 0.013 | 0.687 | 0.000 | 0.000 | 0.011 | 0.001 | 0.000 | 0.032 | 0.000 |

**S4 Table.** Patient 2 Permutation test p-values (FDR corrected) for all the channels across all frequency sub-bands in before-during and before-after state.
