# Supplementary material for: Carbogen inhalation during non-convulsive status epilepticus: A quantitative exploratory analysis of EEG recordings
Source: PLoS One. 2021 Feb 3;16(2):e0240507. doi: 10.1371/journal.pone.0240507 (PMC7857554; doi:10.1371/journal.pone.0240507)
Supplement: S5 Table — (DOCX) [file pone.0240507.s014.docx]

| Channel | Before-During | | | | | Before-After | | | | |
| --- | --- | --- | --- | --- | --- | --- | --- | --- | --- | --- |
|  | **Delta** | **Theta** | **Alpha** | **Beta** | **Gamma** | **Delta** | **Theta** | **Alpha** | **Beta** | **Gamma** |
| 'C3' | 0.26 | 0.35 | 0.30 | 0.16 | 0.34 | 0.24 | 0.25 | 0.22 | 0.07 | -0.26 |
| 'C4' | 0.15 | 0.32 | 0.27 | 0.13 | 0.19 | 0.15 | 0.05 | 0.14 | 0.16 | -0.16 |
| 'CZ' | 0.28 | 0.28 | 0.22 | -0.04 | -0.28 | 0.28 | -0.02 | 0.08 | -0.21 | -0.46 |
| 'F3' | 0.47 | 0.24 | 0.31 | 0.17 | 0.25 | 0.44 | 0.32 | 0.21 | 0.03 | -0.23 |
| 'F4' | 0.22 | 0.22 | 0.41 | 0.24 | -0.03 | 0.05 | 0.21 | 0.17 | 0.20 | -0.32 |
| 'F7' | 0.37 | 0.35 | 0.28 | 0.15 | 0.13 | 0.27 | 0.42 | 0.20 | 0.03 | -0.29 |
| 'F8' | 0.27 | 0.26 | 0.38 | 0.12 | -0.02 | 0.18 | 0.38 | 0.28 | 0.08 | -0.40 |
| 'FZ' | 0.36 | 0.25 | 0.34 | 0.19 | -0.18 | 0.20 | 0.33 | 0.38 | 0.22 | -0.35 |
| 'FP1' | 0.37 | 0.26 | 0.35 | 0.21 | 0.07 | 0.22 | 0.38 | 0.28 | 0.13 | -0.22 |
| 'FP2' | 0.27 | 0.32 | 0.49 | 0.16 | -0.04 | 0.10 | 0.42 | 0.40 | 0.15 | -0.27 |
| 'FPZ' | 0.39 | 0.29 | 0.38 | 0.18 | 0.03 | 0.18 | 0.39 | 0.36 | 0.09 | -0.23 |
| 'O1' | 0.18 | 0.31 | 0.36 | 0.11 | 0.06 | 0.20 | 0.22 | 0.21 | 0.06 | -0.16 |
| 'O2' | 0.18 | 0.35 | 0.35 | 0.15 | 0.15 | 0.22 | 0.23 | 0.25 | 0.08 | -0.15 |
| 'P3' | 0.21 | 0.37 | 0.38 | 0.13 | 0.13 | 0.22 | 0.19 | 0.26 | 0.09 | -0.25 |
| 'P4' | 0.18 | 0.42 | 0.33 | 0.15 | 0.15 | 0.22 | 0.29 | 0.23 | 0.15 | -0.12 |
| 'PZ' | 0.22 | 0.41 | 0.30 | 0.14 | 0.06 | 0.23 | 0.14 | 0.21 | 0.10 | -0.23 |
| 'T3' | 0.36 | 0.34 | 0.21 | 0.14 | 0.16 | 0.26 | 0.33 | 0.17 | 0.06 | -0.18 |
| 'T4' | 0.29 | 0.33 | 0.37 | 0.08 | -0.09 | 0.21 | 0.33 | 0.22 | 0.03 | -0.54 |
| 'T5' | 0.19 | 0.20 | 0.28 | 0.11 | 0.10 | 0.18 | 0.15 | 0.15 | 0.03 | -0.25 |
| 'T6' | 0.26 | 0.33 | 0.41 | 0.19 | 0.19 | 0.22 | 0.23 | 0.23 | 0.02 | -0.20 |

**S5 Table.** Patient 3 Effect size (Effect size *d-*values) for all the channels across all the frequency bands for before-during and before-after state.
