# Supplementary material for: Carbogen inhalation during non-convulsive status epilepticus: A quantitative exploratory analysis of EEG recordings
Source: PLoS One. 2021 Feb 3;16(2):e0240507. doi: 10.1371/journal.pone.0240507 (PMC7857554; doi:10.1371/journal.pone.0240507)
Supplement: S6 Table — (DOCX) [file pone.0240507.s015.docx]

| Channel | Before-During | | | | | Before-After | | | | |
| --- | --- | --- | --- | --- | --- | --- | --- | --- | --- | --- |
|  | **Delta** | **Theta** | **Alpha** | **Beta** | **Gamma** | **Delta** | **Theta** | **Alpha** | **Beta** | **Gamma** |
| 'C3' | 0.090 | 0.030 | 0.047 | 0.294 | 0.030 | 0.180 | 0.156 | 0.185 | 0.664 | 0.133 |
| 'C4' | 0.323 | 0.042 | 0.087 | 0.378 | 0.225 | 0.320 | 0.771 | 0.338 | 0.320 | 0.318 |
| 'CZ' | 0.072 | 0.072 | 0.157 | 0.789 | 0.041 | 0.113 | 0.862 | 0.599 | 0.190 | 0.005 |
| 'F3' | 0.020 | 0.119 | 0.044 | 0.280 | 0.094 | 0.014 | 0.077 | 0.192 | 0.837 | 0.109 |
| 'F4' | 0.162 | 0.167 | 0.022 | 0.119 | 0.854 | 0.775 | 0.187 | 0.275 | 0.209 | 0.051 |
| 'F7' | 0.027 | 0.027 | 0.071 | 0.323 | 0.394 | 0.133 | 0.012 | 0.213 | 0.837 | 0.096 |
| 'F8' | 0.088 | 0.089 | 0.027 | 0.426 | 0.879 | 0.245 | 0.032 | 0.122 | 0.621 | 0.007 |
| 'FZ' | 0.027 | 0.103 | 0.031 | 0.227 | 0.285 | 0.209 | 0.063 | 0.025 | 0.185 | 0.018 |
| 'FP1' | 0.027 | 0.089 | 0.027 | 0.167 | 0.667 | 0.185 | 0.028 | 0.113 | 0.391 | 0.185 |
| 'FP2' | 0.088 | 0.043 | 0.020 | 0.323 | 0.854 | 0.505 | 0.018 | 0.018 | 0.338 | 0.113 |
| 'FPZ' | 0.022 | 0.071 | 0.024 | 0.262 | 0.854 | 0.267 | 0.018 | 0.032 | 0.590 | 0.185 |
| 'O1' | 0.242 | 0.044 | 0.027 | 0.462 | 0.667 | 0.207 | 0.185 | 0.192 | 0.709 | 0.315 |
| 'O2' | 0.250 | 0.027 | 0.027 | 0.323 | 0.344 | 0.185 | 0.185 | 0.156 | 0.603 | 0.320 |
| 'P3' | 0.167 | 0.027 | 0.022 | 0.378 | 0.378 | 0.185 | 0.231 | 0.151 | 0.599 | 0.151 |
| 'P4' | 0.238 | 0.022 | 0.031 | 0.323 | 0.325 | 0.185 | 0.113 | 0.185 | 0.320 | 0.426 |
| 'PZ' | 0.157 | 0.022 | 0.044 | 0.352 | 0.667 | 0.185 | 0.338 | 0.192 | 0.546 | 0.185 |
| 'T3' | 0.029 | 0.032 | 0.176 | 0.361 | 0.294 | 0.143 | 0.063 | 0.278 | 0.721 | 0.254 |
| 'T4' | 0.071 | 0.041 | 0.027 | 0.571 | 0.526 | 0.185 | 0.063 | 0.185 | 0.859 | 0.005 |
| 'T5' | 0.22 | 0.20 | 0.07 | 0.44 | 0.47 | 0.24 | 0.32 | 0.34 | 0.86 | 0.15 |
| 'T6' | 0.09 | 0.04 | 0.02 | 0.22 | 0.22 | 0.19 | 0.19 | 0.19 | 0.87 | 0.21 |

**S6 Table.** Patient 3 Permutation test p-values (FDR corrected) for all the channels across all frequency sub-bands in before-during and before-after state.
