# Supplementary material for: Carbogen inhalation during non-convulsive status epilepticus: A quantitative exploratory analysis of EEG recordings
Source: PLoS One. 2021 Feb 3;16(2):e0240507. doi: 10.1371/journal.pone.0240507 (PMC7857554; doi:10.1371/journal.pone.0240507)
Supplement: S8 Table — (DOCX) [file pone.0240507.s017.docx]

| Channel | Before-During | | | | | Before-After | | | | |
| --- | --- | --- | --- | --- | --- | --- | --- | --- | --- | --- |
|  | **Delta** | **Theta** | **Alpha** | **Beta** | **Gamma** | **Delta** | **Theta** | **Alpha** | **Beta** | **Gamma** |
| 'C3' | 0.009 | 0.002 | 0.004 | 0.708 | 0.905 | 0.680 | 0.035 | 0.001 | 0.116 | 0.085 |
| 'C4' | 0.025 | 0.344 | 0.132 | 0.564 | 0.045 | 0.157 | 0.183 | 0.269 | 0.011 | 0.001 |
| 'CZ' | 0.001 | 0.001 | 0.563 | 0.073 | 0.508 | 0.002 | 0.680 | 0.051 | 0.551 | 0.680 |
| 'F3' | 0.001 | 0.001 | 0.001 | 0.001 | 0.001 | 0.006 | 0.617 | 0.001 | 0.746 | 0.715 |
| 'F4' | 0.073 | 0.433 | 0.142 | 0.866 | 0.563 | 0.679 | 0.001 | 0.151 | 0.303 | 0.011 |
| 'F7' | 0.001 | 0.001 | 0.007 | 0.738 | 0.977 | 0.214 | 0.054 | 0.001 | 0.114 | 0.617 |
| 'F8' | 0.001 | 0.001 | 0.001 | 0.001 | 0.048 | 0.307 | 0.910 | 0.011 | 0.495 | 0.522 |
| 'FP1' | 0.007 | 0.001 | 0.024 | 0.413 | 0.660 | 0.053 | 0.226 | 0.001 | 0.001 | 0.001 |
| 'FP2' | 0.001 | 0.001 | 0.005 | 0.001 | 0.073 | 0.002 | 0.008 | 0.004 | 0.008 | 0.002 |
| 'O1' | 0.977 | 0.059 | 0.036 | 0.977 | 0.800 | 0.008 | 0.042 | 0.003 | 0.007 | 0.014 |
| 'O2' | 0.005 | 0.001 | 0.002 | 0.679 | 0.508 | 0.111 | 0.585 | 0.116 | 0.053 | 0.289 |
| 'P3' | 0.044 | 0.007 | 0.020 | 0.905 | 0.905 | 0.116 | 0.111 | 0.001 | 0.019 | 0.029 |
| 'P4' | 0.001 | 0.013 | 0.003 | 0.564 | 0.905 | 0.026 | 0.715 | 0.822 | 0.157 | 0.910 |
| 'PZ' | 0.096 | 0.001 | 0.081 | 0.470 | 0.977 | 0.326 | 0.116 | 0.011 | 0.007 | 0.052 |
| 'T3' | 0.004 | 0.004 | 0.005 | 0.796 | 0.997 | 0.910 | 0.022 | 0.001 | 0.561 | 0.338 |
| 'T4' | 0.001 | 0.024 | 0.001 | 0.927 | 0.206 | 0.346 | 0.214 | 0.105 | 0.680 | 0.561 |
| 'T5' | 0.336 | 0.034 | 0.079 | 0.996 | 0.734 | 0.052 | 0.007 | 0.001 | 0.226 | 0.071 |
| 'T6' | 0.001 | 0.007 | 0.001 | 0.024 | 0.001 | 0.126 | 0.151 | 0.114 | 0.875 | 0.235 |
| 'C3' | 0.21 | 0.19 | 0.05 | 0.51 | 0.55 | 0.23 | 0.33 | 0.35 | 0.88 | 0.11 |
| 'C4' | 0.07 | 0.03 | 0.00 | 0.21 | 0.21 | 0.16 | 0.13 | 0.15 | 0.90 | 0.20 |

**S8 Table.** Patient 4 Permutation test p-values (FDR corrected) for all the channels across all frequency sub-bands in before-during and before-after state.
