# Supplementary material for: Carbogen inhalation during non-convulsive status epilepticus: A quantitative exploratory analysis of EEG recordings
Source: PLoS One. 2021 Feb 3;16(2):e0240507. doi: 10.1371/journal.pone.0240507 (PMC7857554; doi:10.1371/journal.pone.0240507)
Supplement: S9 Table — (DOCX) [file pone.0240507.s018.docx]

| Channel | Before-During | | | | | Before-After | | | | |
| --- | --- | --- | --- | --- | --- | --- | --- | --- | --- | --- |
|  | **Delta** | **Theta** | **Alpha** | **Beta** | **Gamma** | **Delta** | **Theta** | **Alpha** | **Beta** | **Gamma** |
| 'C4' | 1.09 | 0.86 | 0.54 | 0.23 | -0.14 | 0.90 | 0.96 | 0.45 | 0.07 | 0.03 |
| 'CZ' | 0.91 | 0.87 | 0.77 | 0.54 | -0.17 | 0.68 | 0.66 | 0.02 | -0.44 | -0.60 |
| 'F3' | 0.64 | 0.61 | 0.57 | 0.55 | -0.32 | 0.93 | 1.18 | 1.27 | 0.92 | 0.23 |
| 'F4' | 0.73 | 0.65 | 0.51 | 0.00 | -0.41 | 0.88 | 1.03 | 1.03 | 0.26 | -0.08 |
| 'F7' | 1.00 | 0.56 | 0.45 | 0.31 | -0.25 | 1.11 | 0.89 | 0.73 | 0.24 | 0.09 |
| 'F8' | 1.10 | 0.39 | 0.41 | 0.07 | -0.09 | 1.05 | 1.09 | 1.11 | 0.44 | 0.22 |
| 'FP1' | 0.82 | 0.18 | 0.38 | 0.41 | -0.03 | 0.91 | 0.42 | 0.75 | 0.13 | -0.13 |
| 'FP2' | 0.69 | -0.08 | 0.16 | 0.05 | -0.06 | 0.80 | 0.33 | 0.89 | 0.00 | -0.12 |
| 'O1' | 1.12 | 0.18 | -0.02 | -0.14 | -0.28 | 0.50 | 0.14 | 0.05 | -0.16 | -0.11 |
| 'O2' | 0.51 | 0.80 | 0.61 | 0.57 | 0.12 | 0.40 | 0.31 | 0.29 | -0.10 | -0.16 |
| 'P3' | 0.88 | 0.35 | 0.32 | -0.16 | -0.24 | 0.65 | 0.44 | 0.09 | 0.00 | -0.07 |
| 'P4' | 0.78 | 0.88 | 0.64 | 0.43 | 0.16 | 0.80 | 0.70 | 0.38 | 0.02 | -0.10 |
| 'PZ' | 0.64 | 0.64 | 0.47 | 0.03 | -0.09 | 0.65 | 0.47 | 0.22 | -0.15 | -0.19 |
| 'T3' | 1.15 | 0.61 | 0.30 | -0.03 | -0.57 | 1.06 | 0.80 | 0.35 | 0.14 | 0.21 |
| 'T4' | 1.09 | 0.61 | 0.52 | 0.28 | -0.37 | 0.92 | 1.13 | 0.85 | 0.02 | -0.38 |
| 'T5' | 1.60 | 0.57 | 0.28 | 0.11 | -0.15 | 0.46 | 0.38 | 0.17 | -0.05 | -0.05 |
| 'T6' | 0.58 | 0.74 | 1.02 | 0.07 | -0.38 | 0.76 | 0.87 | 0.75 | 0.27 | 0.06 |
| 'C4' | 0.75 | 0.40 | 0.48 | -0.33 | -0.49 | 0.24 | -0.22 | -0.25 | -0.03 | -0.18 |
| 'CZ' | 0.19 | 0.20 | 0.28 | 0.11 | 0.10 | 0.18 | 0.15 | 0.15 | 0.03 | -0.25 |
| 'F3' | 0.26 | 0.33 | 0.41 | 0.19 | 0.19 | 0.22 | 0.23 | 0.23 | 0.02 | -0.20 |

**S9 Table.** Patient 5 Effect size (Cohen’s *d*–values) for all the channels across all the frequency bands for before-during and before-after state.
