# Supplementary material for: Carbogen inhalation during non-convulsive status epilepticus: A quantitative exploratory analysis of EEG recordings
Source: PLoS One. 2021 Feb 3;16(2):e0240507. doi: 10.1371/journal.pone.0240507 (PMC7857554; doi:10.1371/journal.pone.0240507)
Supplement: S10 Table — (DOCX) [file pone.0240507.s019.docx]

| Channel | Before-During | | | | | Before-After | | | | |
| --- | --- | --- | --- | --- | --- | --- | --- | --- | --- | --- |
|  | **Delta** | **Theta** | **Alpha** | **Beta** | **Gamma** | **Delta** | **Theta** | **Alpha** | **Beta** | **Gamma** |
| 'C4' | 0.000 | 0.000 | 0.000 | 0.055 | 0.380 | 0.000 | 0.000 | 0.001 | 0.705 | 0.894 |
| 'CZ' | 0.000 | 0.000 | 0.000 | 0.000 | 0.246 | 0.000 | 0.000 | 0.912 | 0.000 | 0.000 |
| 'F3' | 0.000 | 0.000 | 0.000 | 0.000 | 0.017 | 0.000 | 0.000 | 0.000 | 0.000 | 0.117 |
| 'F4' | 0.000 | 0.000 | 0.000 | 0.996 | 0.000 | 0.000 | 0.000 | 0.000 | 0.082 | 0.640 |
| 'F7' | 0.000 | 0.000 | 0.001 | 0.003 | 0.074 | 0.000 | 0.000 | 0.000 | 0.105 | 0.610 |
| 'F8' | 0.000 | 0.006 | 0.002 | 0.915 | 0.687 | 0.000 | 0.000 | 0.000 | 0.000 | 0.137 |
| 'FP1' | 0.000 | 0.205 | 0.005 | 0.000 | 0.888 | 0.000 | 0.002 | 0.000 | 0.459 | 0.428 |
| 'FP2' | 0.000 | 0.593 | 0.261 | 0.977 | 0.880 | 0.000 | 0.022 | 0.000 | 0.992 | 0.481 |
| 'O1' | 0.000 | 0.206 | 0.910 | 0.380 | 0.000 | 0.001 | 0.357 | 0.763 | 0.330 | 0.547 |
| 'O2' | 0.000 | 0.000 | 0.000 | 0.000 | 0.429 | 0.003 | 0.032 | 0.045 | 0.558 | 0.301 |
| 'P3' | 0.000 | 0.006 | 0.015 | 0.312 | 0.032 | 0.000 | 0.002 | 0.601 | 0.992 | 0.706 |
| 'P4' | 0.000 | 0.000 | 0.000 | 0.000 | 0.329 | 0.000 | 0.000 | 0.004 | 0.926 | 0.558 |
| 'PZ' | 0.000 | 0.000 | 0.001 | 0.933 | 0.722 | 0.000 | 0.001 | 0.137 | 0.357 | 0.212 |
| 'T3' | 0.000 | 0.000 | 0.032 | 0.895 | 0.000 | 0.000 | 0.000 | 0.012 | 0.387 | 0.167 |
| 'T4' | 0.000 | 0.000 | 0.000 | 0.037 | 0.005 | 0.000 | 0.000 | 0.000 | 0.928 | 0.007 |
| 'T5' | 0.000 | 0.000 | 0.044 | 0.505 | 0.358 | 0.001 | 0.007 | 0.275 | 0.796 | 0.796 |
| 'T6' | 0.000 | 0.000 | 0.000 | 0.814 | 0.000 | 0.000 | 0.000 | 0.000 | 0.062 | 0.763 |
| 'C4' | 0.000 | 0.005 | 0.001 | 0.018 | 0.000 | 0.117 | 0.139 | 0.101 | 0.894 | 0.235 |
| 'CZ' | 0.19 | 0.17 | 0.04 | 0.45 | 0.49 | 0.23 | 0.34 | 0.36 | 0.90 | 0.09 |
| 'F3' | 0.06 | 0.02 | 0.00 | 0.19 | 0.18 | 0.15 | 0.12 | 0.14 | 0.91 | 0.19 |

**S10 Table.** Patient 5 Permutation test p-values (FDR corrected) for all the channels across all frequency sub-bands in before-during and before-after state.
