# Supplementary material for: Carbogen inhalation during non-convulsive status epilepticus: A quantitative exploratory analysis of EEG recordings
Source: PLoS One. 2021 Feb 3;16(2):e0240507. doi: 10.1371/journal.pone.0240507 (PMC7857554; doi:10.1371/journal.pone.0240507)
Supplement: S11 Table — (DOCX) [file pone.0240507.s020.docx]

| Patient ID | Broadband Power | | Functional Connectivity | | Path Length | | Clustering Coefficient | |
| --- | --- | --- | --- | --- | --- | --- | --- | --- |
|  | ***Before-During*** | ***Before-After*** | ***Before-During*** | ***Before-After*** | ***Before-During*** | ***Before-After*** | ***Before-During*** | ***Before-After*** |
|  |  |  |  |  |  |  |  |  |
| Patient 1 | 0.38 | -0.67 | -0.04 | -0.26 | 0.05 | 0.26 | -0.05 | -0.22 |
| Patient 2 | -1.11 | -0.88 | -0.60 | -0.80 | 0.61 | 0.72 | -0.39 | -0.63 |
| Patient 3 | 0.32 | -0.17 | 0.03 | 0.31 | -0.09 | -0.36 | -0.05 | 0.27 |
| Patient 4 | -0.01 | -0.10 | 0.25 | 0.42 | -0.14 | -0.34 | 0.28 | 0.46 |
| Patient 5 | -0.14 | 0.01 | 0.82 | 0.42 | -0.88 | -0.41 | 0.81 | 0.39 |

**S11 Table.** Effect size (Cohen’s *d*–values) for broadband power time series, functional connectivity time series, path length time series, and clustering coefficient time series.
