# Supplementary material for: Carbogen inhalation during non-convulsive status epilepticus: A quantitative exploratory analysis of EEG recordings
Source: PLoS One. 2021 Feb 3;16(2):e0240507. doi: 10.1371/journal.pone.0240507 (PMC7857554; doi:10.1371/journal.pone.0240507)
Supplement: S12 Table — (DOCX) [file pone.0240507.s021.docx]

| Patient ID | Broadband Power | | Functional Connectivity | | Path Length | | Clustering Coefficient | |
| --- | --- | --- | --- | --- | --- | --- | --- | --- |
|  | ***Before-During*** | ***Before-After*** | ***Before-During*** | ***Before-After*** | ***Before-During*** | ***Before-After*** | ***Before-During*** | ***Before-After*** |
|  |  |  |  |  |  |  |  |  |
| Patient 1 | 0.00 | 0.00 | 0.82 | 0.06 | 0.669 | 0.064 | 0.716 | 0.110 |
| Patient 2 | 0.00 | 0.00 | 0.00 | 0.00 | 0.000 | 0.000 | 0.007 | 0.000 |
| Patient 3 | 0.03 | 0.30 | 0.82 | 0.03 | 0.509 | 0.011 | 0.716 | 0.054 |
| Patient 4 | 0.96 | 0.57 | 0.06 | 0.00 | 0.343 | 0.017 | 0.050 | 0.001 |
| Patient 5 | 0.47 | 0.96 | 0.00 | 0.00 | 0.000 | 0.0045 | 0.000 | 0.007 |

**S12 Table.** Permutation test p-values for broadband band power time series, functional connectivity time series, path length time series, and clustering coefficient time series.
