# Supplementary material for: Carbogen inhalation during non-convulsive status epilepticus: A quantitative exploratory analysis of EEG recordings
Source: PLoS One. 2021 Feb 3;16(2):e0240507. doi: 10.1371/journal.pone.0240507 (PMC7857554; doi:10.1371/journal.pone.0240507)
Supplement: Attachment — Submitted filename: Response To reviewers.docx [file pone.0240507.s022.docx]

**Reviewer #1:**

This is a report of the study on carbogen inhalation during Non-Convulsive Status Epilepticus: A quantitative analysis of EEG recordings.
Below are some comments:

We thank the reviewer for their review and comments which we address below.

- The objective of the study was to ‘quantify the effect of inhaled 5% carbon-dioxide/95% oxygen on EEG recordings from patients in non-convulsive status epilepticus (NCSE)’. The objective was however, not followed by a precise/clear hypothesis.

The hypothesis is now added to the manuscript in the introduction. “We hypothesised quantitative EEG changes during- and after-carbogen administration.”

Neither did the authors mention how the sample size of 5 patients derived (no description on how the study was powered, and no description on what was the targeted effect size).

We used all data from all patients with pre- during- and post-carbogen administration available from Forsyth et al (2016), which contains further details pertaining to study power. We wish to highlight that the 2016 study was originally to evaluate the safety and efficacy of low dose carbogen administration. Our present study is an opportunistic retrospective analysis of that unique and rare prospective clinical trial data.

- In addition to small sample size of 5 patients, patients enrolled in the study had quite diverse etiology and hence introducing heterogeneity in the selected population. This raises some questions on the usefulness of the current analysis

We acknowledge the reviewer’s comment. We include the following in discussion.

“Other factors for the ineffectiveness of carbogen might be, first, the lack of “aggressiveness” (concentration/duration), second, the heterogeneity of the underlying aetiology of the patients, and third our limited sample size.”

- Patients selection was retrospective and this added heterogeneity in the selected samples/patients. Normally in such a retrospective study, larger sample size is expected to compensate for the heterogeneity due to not being able to make a proper selection of patients. Again this restriction in addition to very small sample size should be explained and addressed.

The following is now added to the manuscript.

“Patient recruitment in this prospective trial was slow and despite recruiting from other centres a sample of only five patients was possible.”
- Restriction of the retrospective analysis also applies in the treatment onset, as there were indications that some patients may receive the treatment (carbogen) in different time frame (onset) than other. This adds further heterogeneity in the collected data.

Agreed, we acknowledge this as a one of the limitations of this study. This was a prospective trial with a novel drug which hasn’t been used in this circumstance so we presented results in a cautious manner.

- Lacking of control arm (i.e. patients not receiving any treatment) in this analysis make the data interpretation is difficult. Even though the nature of the data is longitudinal/time series in which some parts in each patient exposed to treatment and the rest not, which implies that patient can become their own control for different phases they involved during the study, but in the event of inconsistent results as currently observed, the existence of non-exposed patients might have helped the interpretation.

We appreciate the concerns in the interpreting the data and therefore we present the results as in an exploratory fashion. We used each patient’s non-exposed time frame as control signal. We acknowledge the reviewer’s suggestion of using non-exposed patients as controls, however, the outcome of current analysis states that except one patient, remaining cohort does not show any effect on EEG.

- The time series analysis may have quite large data on EEG collected longitudinally, yet the experimental unit of this study is patient. With this restriction, it is difficult if possible at all to make any conclusion from this analysis.

We acknowledge with the reviewer’s comment. We did perform the analysis on individual EEG channels across three phases (before, during and after phases of carbogen inhalation) and can be found in supplementary materials. The results are not drastically different from the patient level results in terms of inconsistency.

We do think it is reasonable to conclude that we found no evidence to support the hypothesis that 5% carbogen administration is associated with large and consistent alterations to commonly used quantitative EEG metrics in this patient cohort.

- Page 8: ‘Because final confirmation of eligibility required confirmation of NCSE on EEG, written “consent in principle” to participate in the study was obtained with final verbal consent to proceed once NCSE was confirmed electrographically’. How many patients were screened out and declared ineligible?

There was one child in whom NCSE was suspected (on basis of known seizure disorder and parental report of reduced alertness and interaction) but who in fact turned out not to be in NCSE once the EEG was in process and was therefore subsequently excluded.

**Reviewer #2:**

Manuscript number: PONE-D-19-31394

Reviewer’s Comments:
The manuscript presents a quantitative analysis of EEG recordings of carbogen inhalation during NCSE. The study described in this manuscript is interesting but the following points need to be addressed:

We thank the reviewer for their review and comments which we address below.

1) quantitative characterization should be performed in relation to outcome

In terms of clinical outcome all patients did not respond favourably (i.e. seizure persistence despite carbogen administration). In terms of quantitative EEG changes, which was our variable of interest has shown large effect on patient 5’s EEG (suppression of bandpower in lower frequency bands) but has no effect of rest of the cohort’s EEG.

2) Ethics approval for the retrospective study should be obtained

We have now included the ethical approval reference in the text (Ref: 1804/2020).

3) inclusion and exclusion criteria are missed

The following is now added to the manuscript.

“Inclusion criteria included (i) confirmed NCSE, with EEG manifestation, and (ii) reduced awareness or function confirmed by a parent or carer. Patients requiring other urgent treatment, or patients with capillary pCO2>8kPa were excluded.”

4) EEG procedures should be added

We have included EEG acquisition information including sampling rate and electrode numbers in Table 1.

5) Statistical analysis: please specify which tests were applied for comparisons and the statistical software used

Two statistical tests were applied for the comparisons: Permutation test (10000 permutations; mean) and Cohen’s *d*. Detailed information can be found in Statistical Analysis section.

MATLAB was used for the above-mentioned statistical tests. This is now added to the Statistical Analysis section.
