# Supplementary material for: Carbogen inhalation during non-convulsive status epilepticus: A quantitative exploratory analysis of EEG recordings
Source: PLoS One. 2021 Feb 3;16(2):e0240507. doi: 10.1371/journal.pone.0240507 (PMC7857554; doi:10.1371/journal.pone.0240507)
Supplement: Attachment — Submitted filename: Response To Reviewers.docx [file pone.0240507.s023.docx]

Reviewers' comments:

Reviewer's Responses to Questions

**Comments to the Author**

1. If the authors have adequately addressed your comments raised in a previous round of review and you feel that this manuscript is now acceptable for publication, you may indicate that here to bypass the “Comments to the Author” section, enter your conflict of interest statement in the “Confidential to Editor” section, and submit your "Accept" recommendation.

Reviewer #1: (No Response)

Reviewer #2: (No Response)

2. Is the manuscript technically sound, and do the data support the conclusions?

The manuscript must describe a technically sound piece of scientific research with data that supports the conclusions. Experiments must have been conducted rigorously, with appropriate controls, replication, and sample sizes. The conclusions must be drawn appropriately based on the data presented.

Reviewer #1: Partly

Reviewer #2: Partly

3. Has the statistical analysis been performed appropriately and rigorously?

Reviewer #1: Yes

Reviewer #2: No

4. Have the authors made all data underlying the findings in their manuscript fully available?

The [PLOS Data policy](http://www.plosone.org/static/policies.action#sharing) requires authors to make all data underlying the findings described in their manuscript fully available without restriction, with rare exception (please refer to the Data Availability Statement in the manuscript PDF file). The data should be provided as part of the manuscript or its supporting information, or deposited to a public repository. For example, in addition to summary statistics, the data points behind means, medians and variance measures should be available. If there are restrictions on publicly sharing data—e.g. participant privacy or use of data from a third party—those must be specified.

Reviewer #1: Yes

Reviewer #2: Yes

5. Is the manuscript presented in an intelligible fashion and written in standard English?

PLOS ONE does not copyedit accepted manuscripts, so the language in submitted articles must be clear, correct, and unambiguous. Any typographical or grammatical errors should be corrected at revision, so please note any specific errors here.

Reviewer #1: Yes

Reviewer #2: Yes

6. Review Comments to the Author

Please use the space provided to explain your answers to the questions above. You may also include additional comments for the author, including concerns about dual publication, research ethics, or publication ethics. (Please upload your review as an attachment if it exceeds 20,000 characters)

Reviewer #1: Thank you for the reply. Below please find some further comments in a couple of points:

- Lacking of control arm (i.e. patients not receiving any treatment) in this analysis make the data interpretation is difficult. Even though the nature of the data is longitudinal/time series in which some parts in each patient exposed to treatment and the rest not, which implies that patient can become their own control for different phases they involved during the study, but in the event of inconsistent results as currently observed, the existence of non-exposed patients might have helped the interpretation.

A: We appreciate the concerns in the interpreting the data and therefore we present the results as in an exploratory fashion. We used each patient’s non-exposed time frame as control signal. We acknowledge the reviewer’s suggestion of using non-exposed patients as controls, however, the outcome of current analysis states that except one patient, remaining cohort does not show any effect on EEG.

**Re: The statement that the study is exploratory in nature is an important one and should be clearly mentioned in the manuscript**

***Authors:*** *We agree with the reviewer’s suggestion. We now added “explored” in the last paragraph of introduction section (In this exploratory study we investigated the effect of carbogen….)*

*We have also updated the title to:*

*Carbogen inhalation during Non-Convulsive Status Epilepticus: A quantitative* ***exploratory*** *analysis of EEG recordings*

- The time series analysis may have quite large data on EEG collected longitudinally, yet the experimental unit of this study is patient. With this restriction, it is difficult if possible at all to make any conclusion from this analysis.

A: We acknowledge with the reviewer’s comment. We did perform the analysis on individual EEG channels across three phases (before, during and after phases of carbogen inhalation) and can be found in supplementary materials. The results are not drastically different from the patient level results in terms of inconsistency.
We do think it is reasonable to conclude that we found no evidence to support the hypothesis that 5% carbogen administration is associated with large and consistent alterations to commonly used quantitative EEG metrics in this patient cohort.

**Re: Hypothesis did not specify how large the changes/alterations were sought. With this non-specific hypothesis, the conclusion was also blurry and not specific. Therefore, the above stated conclusion is not fully supported. In your above conclusion, how do you define ‘large and consistent alterations’? This is particularly a concern since the sample size of the study was only 5 patients.**

***Authors:*** We have now been more precise in our hypothesis in the last paragraph of the introduction.

*“In this exploratory study we investigated the effect of carbogen on band power and functional connectivity across five frequency sub-bands (delta, theta, alpha, beta and gamma). We hypothesised medium to large (cohen’s d >0.5) quantitative EEG changes during- and after-carbogen administration.”*

**Reviewer #2: Please clearly indicate in the methods section the study design and that data are available from the study of Forsyth and collaborators (2016) with a brief description**

***Authors:*** As reviewer suggested we now added the following sentence at the beginning of the Methods section. “The study design and the data are available from Forsyth et al (2016) as this is the follow up study”.

**Please add a brief description of the sample size calculation**

***Authors:*** *The following paragraph is added in Patient information and recordings of Methods section*

*“Patient recruitment in the prospective trail was very slow despite opening the recruitment from additional centres. The recruitment was closed by Trial steering committee 30 months after recruiting the first child. This is done on the basis that substantial increases in recruitment rates were unrealistic. Forsyth et al (2016) recruited six subjects, however, quality EEG recordings are available only for five of them”.* **Please add and discuss in the discussion the lack of the control arm**

*We have now included this in the limitations paragraph of discussion:*

*“…without a control arm of non-delivery of carbogen.”*

7. PLOS authors have the option to publish the peer review history of their article ([what does this mean?](https://journals.plos.org/plosone/s/editorial-and-peer-review-process#loc-peer-review-history)). If published, this will include your full peer review and any attached files.


If you choose “no”, your identity will remain anonymous but your review may still be made public.


**Do you want your identity to be public for this peer review?** For information about this choice, including consent withdrawal, please see our [Privacy Policy](https://www.plos.org/privacy-policy).

Reviewer #1: No

Reviewer #2: No

[NOTE: If reviewer comments were submitted as an attachment file, they will be attached to this email and accessible via the submission site. Please log into your account, locate the manuscript record, and check for the action link "View Attachments". If this link does not appear, there are no attachment files.]

While revising your submission, please upload your figure files to the Preflight Analysis and Conversion Engine (PACE) digital diagnostic tool, <https://pacev2.apexcovantage.com/>. PACE helps ensure that figures meet PLOS requirements. To use PACE, you must first register as a user. Registration is free. Then, login and navigate to the UPLOAD tab, where you will find detailed instructions on how to use the tool. If you encounter any issues or have any questions when using PACE, please email PLOS at [figures@plos.org](mailto:figures@plos.org). Please note that Supporting Information files do not need this step.
